# When you know better, do better: a reconceptualization of SEL for Black youth

**DOI:** 10.3389/fpsyg.2026.1844241

**Published:** 2026-06-18

**Authors:** Kamontá Heidelburg, Meagan Scott, Tai A. Collins

**Affiliations:** 1 Department of Educational Studies, The Ohio State University, Columbus, OH, United States; 2 Department of Educational, School, and Counseling Psychology, University of Kentucky, Louisville, KY, United States; 3 University of Cincinnati, Cincinnati, OH, United States

**Keywords:** advocacy, Black students, culturally responsive, SEL, wellbeing

## Abstract

Social-emotional learning (SEL) is often presented as a way to promote positive student outcomes. However, current SEL programs may reflect Eurocentric, heterosexual, ableist, middle-class American values and overlook the needs of racially/ethnically minoritized youth. To help school psychologists engage in social justice advocacy to improve Black students' wellbeing, this conceptual article highlights five key components of SEL for Black youth. The authors focus on: (1) afrocentrism; (2) anti-oppressive frameworks; (3) a strength-based approach; (4) student empowerment and healing; and (5) evidence-based practices as essential to SEL to promote positive academic, behavioral, and social-emotional outcomes for Black youth. Supporting research for each component is included, along with recommendations for system-level integration to enhance school interventions, promote community partnerships, and establish policies that meet the needs of Black youth.

## 1 Introduction

In a time where civil rights and liberties are being stripped away, attending school can be challenging for Black students, as they often find themselves in educational environments that are not physically, mentally, or emotionally safe (Voight et al., 2015). The United States educational system has a history of perpetuating racial violence against Black students and those from marginalized backgrounds (Boutte and Bryan, 2021; Simson, 2014). School-based violence directed at Black students takes many forms: (1) viewing Black students as less capable compared to peers of other races (Boutte and Bryan, 2021); (2) school personnel being complicit with systemic policies that often reflect anti-Black ideologies (e.g., zero-tolerance disciplinary policies, overrepresentation in special education; Dumas and Ross, 2016); and (3) delivering curricula that lack cultural responsiveness (Johnson et al., 2019). These actions, whether intentional or not, convey to Black youth that their lives are devalued and unprotected, which negatively impacts their sense of belonging, motivation, mental health, and identity (Congressional Black Caucus, 2019; Dumas and Ross, 2016; Johnson, 2016; Johnson et al., 2019).

## 2 The need for culturally responsive SEL

Social-emotional learning (SEL) can effectively enhance students' academic and behavioral outcomes. A range of short- and long-term student outcomes are associated with SEL interventions, including improved academic achievement, self-awareness, responsible decision-making, self-image, relationship skills, mental health, social awareness, prosocial behavior, and decreased antisocial behavior, substance abuse, as well as symptoms of anxiety, stress, and depression (McCallops et al., 2019; Sklad et al., 2012; Taylor et al., 2017). While there is substantial evidence supporting the effectiveness of SEL in promoting positive student outcomes, current SEL interventions may not be culturally responsive to marginalized students' identities, including race, sexual orientation, gender, disability, and socioeconomic status (Rowe and Trickett, 2018). Several educators and scholars have criticized the readily available color-evasive SEL interventions for reflecting Eurocentric, heterosexual, middle-class, and able-bodied beliefs and values (Camangian and Cariaga, 2021; Drake and Oglesby, 2020; Jones et al., 2026; Mayes et al., 2022; McCall et al., 2023). Rowe and Trickett (2018) conducted an analysis of 117 published school-based universal SEL intervention studies in terms of their attention to student diversity characteristics (e.g., gender, race and ethnicity, socioeconomic status (SES), disability, and sexual orientation, and gender identity). Researchers found that many articles in their review did not report any demographic information. Furthermore, studies with reported demographics showed variability in effects across students' characteristics. The lack of reported demographic data and the considerable variability in effects across diversity characteristics in SEL research greatly limit the ability to generalize SEL intervention results across student groups, ultimately hindering the determination of which interventions are evidence-based for specific student populations.

Additionally, when students' cultural identities are not centered or reported in psychological research and practice, SEL interventions may neglect students' cultural differences and the historical and social influences that sustain oppressive systems of power and privilege (Mayes et al., 2022; McCall et al., 2023; Rowe and Trickett, 2018). When SEL interventions neglect students' cultural identities, SEL can be misused as a tool to enforce compliance and control, often penalizing racially and ethnically minoritized (REM) students by upholding standards of whiteness. This ultimately reinforces racial hierarchy and oppression and contributes to the hyper-surveillance that Black students may face in schools. Consequently, SEL interventions can potentially exacerbate ongoing racial trauma and the negative outcomes for Black students, which undermines the goal of SEL (Camangian and Cariaga, 2021; Mikhail et al., 2018).

Such criticisms are valid given that the United States education system is a microcosm of society where the standards, policies, and practices in schools are based on tenets of white supremacist ideologies used to sustain racism, a system of oppression based on race (McCall et al., 2023). Therefore, any school curriculum, policy, or practice that is not intentional in combating racism, bias, and oppression can be used as a weapon to oppress Black students. Lin et al. (2023) highlighted potential risks and harm with SEL implementation in schools, noting the potential of white teachers retraumatizing Black, Brown, and Queer youth and unknowingly stripping students of their culture, language, and identity by oppressing their bodies, clothing choices, and spoken language. For Black students, implementing culturally irrelevant SEL is one type of anti-Black violence in schools (Boutte and Bryan, 2021). When SEL practices apply a colorevasive approach that refuses to acknowledge race or other aspects of students' identities, SEL competencies may be rooted in whiteness and used to enforce a limited set of values and beliefs on Black youth (Drake and Oglesby, 2020). Therefore, existing SEL can do more harm than good for Black students if SEL interventions are not culturally responsive to Black students' experiences and needs.

Since Black students experience persistent racial trauma due to various oppressive policies and practices (Mikhail et al., 2018), SEL must address the historical and social influences that create and maintain oppressive systems and equip Black students with skills to regulate their emotions and prioritize their wellbeing within oppressive systems rather than merely coping through the pain of oppression (Drake and Oglesby, 2020; Mayes et al., 2022; McCall et al., 2023; Simmons, 2021). Furthermore, SEL must not overlook the cultural, social, and political realities of Black students or embrace a race-neutral perspective. Current SEL interventions may adopt a deficit perspective and assume that everyone's emotions and expressions are the same, promoting a one-size-fits-all approach that ignores the impact of systemic oppression on historically marginalized students. A race-neutral approach in SEL often centers whiteness, perpetuating an oppressive, racist system (Lin et al., 2023). Claiming not to “see race” indicates that educators and researchers fail to acknowledge racial inequalities and injustices present in schools. When SEL interventions neglect the historical and social factors that uphold oppressive systems, Black students are deprived of the social and emotional skills necessary to navigate and overcome challenging educational environments (Camangian and Cariaga, 2021). Insufficient understanding of Black students' lived experiences leads to SEL interventions that fail to address ongoing racial trauma. SEL interventions that fail to address the sociopolitical contexts that impact Black students and combat racial and social injustice will not prepare Black children to navigate our complex, unjust society. As Simmons (2021) noted, SEL then risks becoming “white supremacy with a hug” (n.p.). This hug can murder the spirit of Black students by minimizing their positive outcomes in schools. Given the potential harm of current SEL interventions, advancing SEL requires attention to students' cultural identities and a reconceptualization that centers Black students' liberation.

## 3 Culturally responsive SEL

Jagers et al. (2018, 2019) highlighted the importance of transformative SEL, which promotes system critique and aims to advance social justice by preparing justice-oriented citizens through the development of core competencies such as identity, agency, belonging, curiosity, and collaborative problem-solving to address the various inequities created by systems of oppression. For Black students, transformative SEL must deliberately analyze and dismantle oppressive structures that demand people to assimilate to whiteness and comply with oppressive conditions (Simmons, 2021). Hence, culturally responsive SEL offers a transformative approach to advancing SEL and meeting the needs of REM youth.

Culturally responsive SEL closely aligns with Jagers et al.'s (2018) transformative approach to SEL. Culturally responsive SEL involves utilizing information about specific cultural groups (e.g., race, disability, sexual orientation, gender, religion, social class, and age) along with an individual's lived experiences to develop, revise, inform, and adapt SEL interventions, making them more effective for a given population (Bernal et al., 1995). Several researchers have indicated that culturally responsive SEL can enhance numerous positive prosocial and protective factors for youth (Albritton et al., 2024; Brown et al., 2018; Li et al., 2025; Lim et al., 2026). For Black students in particular, research suggests several positive prosocial outcomes associated with culturally responsive SEL, including racial/ethnic identity, self-esteem, and prosocial behaviors, as well as reductions in inappropriate behaviors (Heidelburg et al., 2025). Furthermore, culturally responsive SEL can help Black students avoid disciplinary exclusion. For example, Campbell et al. (2023) examined the impact of a culturally adapted SEL curriculum on externalizing behaviors among Black male learners in an urban elementary school. They discovered reduced externalizing problem behaviors and increased social and emotional competencies.

Although culturally responsive SEL serves as the overarching approach to supporting all culturally and ethnically marginalized students, it is crucial that school-based SEL implementation intentionally addresses the specific needs of the student population it aims to serve. For example, for REM students, Lin et al. (2023) noted that in-service educators suggested that SEL can serve as a praxis that facilitates healing, responds to student and community needs, and emphasizes humanity alongside racial and social justice. To better serve REM students, adopting an antiracist perspective in SEL can help further culturally responsive SEL.

### 3.1 Antiracist SEL

An antiracist lens to SEL centers on protecting REM students from dehumanizing conditions inside and outside of school by promoting freedom and justice through empowerment, hope, and wellbeing (Mayes et al., 2022). An antiracist lens embraces the unique and diverse cultures, backgrounds, and lived experiences and centers the humanity and brilliance of REM students. An antiracist lens offers an honest interrogation of existing SEL and aids in developing antiracist policies and practices to support REM students' school success. Mayes et al. (2022) offer an Antiracist Social Emotional Justice Learning approach (ASEJL) that outlines five principles for educators to apply to SEL: (1) critical theoretical frameworks; (2) anti-bias building blocks; (3) student and family voice; (4) strengths-based empowerment; and (5) homeplace. These five principles encompass critical theories relevant to REM youth development (critical theoretical frameworks) and aid in developing critical consciousness (anti-bias building blocks), which serves as the foundation of ASEJL. ASEJL focuses on identity, diversity, justice, and action through strengths-based empowerment, which necessitates an intentional shift from deficit-oriented views of REM students to a focus on REM students' and families' voices (student and family voice and strengths-based empowerment) (Mayes et al., 2022). ASEJL also promotes safe spaces for REM students (homeplace). Safe spaces for REM students mean creating space for REM students to process the harm from experiencing persistent racial trauma and to combat variousisms (e.g., racism, classism, ableism, and sexism) and other forms of oppression found in schools.

### 3.2 Liberatory SEL

Although using an antiracist approach can help propel culturally responsive SEL for REM students, it is essential to note that antiracist does not necessarily mean anti-black or anti-oppressive for Black people. With an antiracist approach that is focused on REM students as a whole (i.e., ASEJL approach), oppressive anti-Black policies and practices involving SEL that harm Black youth may be neglected, ignored, or hidden. For SEL to be culturally responsive to the needs of Black youth specifically, SEL must intentionally avoid anti-blackness and promote dismantling all systemic, institutional, and individual practices that harm Black youth and impede their social-emotional wellbeing. To specifically meet the needs of Black youth, a liberatory focus should be adopted for school-based SEL implementation. Liberation allows Black people to be free to live, learn, and thrive in the comfort of their existence (Love, 2019). A liberatory approach to SEL for Black youth centers on humanizing Black students and equipping them with tools to navigate and dismantle the oppressive systems they encounter (Camangian and Cariaga, 2021). A liberatory approach to SEL can aid Black students in recognizing and analyzing the systems of oppression that impede them (Lin et al., 2023). A liberatory approach to SEL for Black youth seeks to mitigate the effects of systemic racist policies and practices by promoting culturally responsive, strength-based SEL that affirms Black students and their experiences, centers their needs, and protects them (Drake and Oglesby, 2020). Additionally, liberatory SEL helps educators see students as complete human beings, including their multifaceted identities shaped by the intersectional systems of privilege and oppression that shape their lives (Love, 2019).

## 4 Components of SEL for Black youth

**Figure 1:**
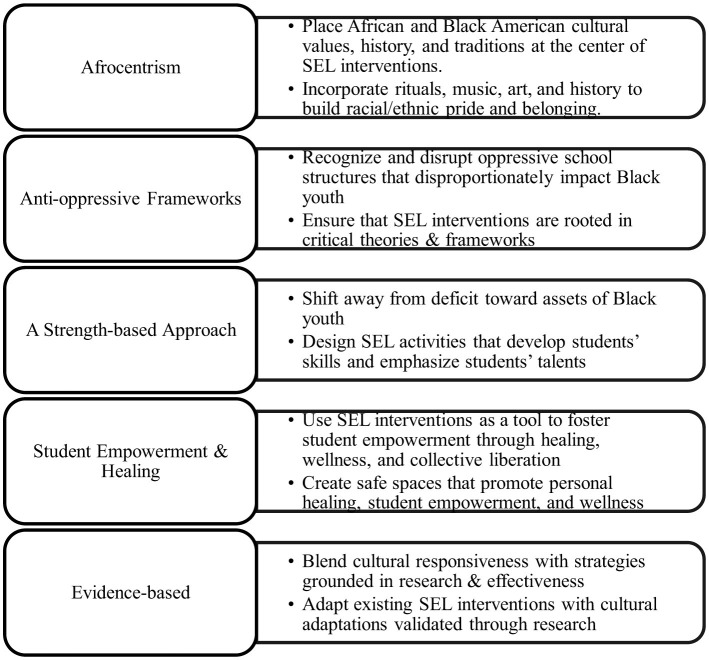
Summary of foundational components of SEL for Black youth.

For Black youth, SEL can no longer be practiced as skill development without centering humanity, healing, liberation, and social justice that recognize, honor, and affirm students' identities, realities, and lived experiences. To broaden school advocacy efforts and the ASEJL approach toward an intentional focus on Black youth, five essential elements for implementing school-based SEL are provided to enhance the positive development and wellbeing of Black youth. These five components: (1) afrocentrism; (2) anti-oppressive frameworks; (3) strength-based approach; (4) student empowerment and healing; and (5) evidence-based practices serve as foundational elements to better meet the needs of Black students. These components offer a means to liberatory SEL for Black youth and are informed by our practice within school psychology and extensive research on positive Black youth development. The following section will examine these five components with supporting research and provide suggestions for implementing them at the system level to improve individual interventions, strengthen community partnerships, and develop systemic policies that address the needs of Black youth. Figure 1 summarizes the main ideas and practices related to each of the five components.

### 4.1 Afrocentrism

Afrocentrism, also known as “Afrocentric” or “African-centered,” is a framework for culturally responsive positive youth development that focuses on instilling indigenous African and African diasporan cultural beliefs, values, ideologies, and practices among Black youth (Asante, 2003; Lateef et al., 2022). Afrocentricism centers on Black people and their experiences to challenge white supremacist ideology and epistemology (Asante, 2003). Afrocentricism challenges systemic barriers such as racism, inadequate resources, and discrimination within oppressive institutional systems (Asante, 2003; Belgrave and Brevard, 2015). Given the benefits of Afrocentrism for Black youth, educators and scholars within the Black community have increasingly advocated for the use of Afrocentric-based policies, practices, and culturally responsive SEL interventions for Black youth (Belgrave and Brevard, 2015; Lateef et al., 2022).

Afrocentric interventions aim to help Black youth enhance their sense of belonging, self-worth, racial and ethnic pride, wellbeing, and appreciation of Black Americans and people of the African diaspora, as well as their resistance to and resilience against oppression (Belgrave and Brevard, 2015; Lateef and Anthony, 2020). Afrocentric interventions are positively associated with many outcomes for Black children, including relationship skills, identity empowerment, positive choice-making, self-esteem, ethnic/racial identity, prosocial behaviors, less exclusionary discipline, and mental health (Lateef et al., 2022; Loyd and Williams, 2017).

Integrating Afrocentrism into school policies and practices, such as SEL, is critical for supporting the development of Black youth (Asante, 2003). One common way to integrate Afrocentrism into SEL is to use the value system of *Nguzo Saba* as a theoretical framework for SEL interventions. Maulana Karenga (1988), a prominent African-American scholar, suggested that the seven principles of *Nguzo Saba*: *Umoja* (unity); *Ujima* (collective work and responsibility); *Nia* (purpose); *Kujichagulia* (self-determination); *Ujamaa* (cooperative economics); *Kuumba* (creativity); and *Imani* (faith) are the “minimum set of values” Black people need to promote positive outcomes (Karenga, 1988, p. 43). Therefore, centering the principles of *Nguzo Saba* to support culturally responsive SEL interventions for Black students is an effective means of increasing a range of protective factors that help Black youth navigate oppressive systems and anti-Black policies and practices (see Jones et al. (2023) for more on centering the principles of *Nguzo Saba* to improve school outcomes for Black males). *Sisters of Nia* (Belgrave et al., 2008) and *Brothers of Ujima* (Belgrave et al., 2011) are two well-known Afrocentric programs that use the principles of *Nguzo Saba* to support Black girls and Black males. Several scholars have found increased social, emotional, behavioral, and academic outcomes for both interventions, with Black children including increases in ethnic identity, self-concept, and Africentric values (Belgrave et al., 2004; Graves and Aston, 2018; Jones and Lee, 2020).

Afrocentrism is essential to ensuring SEL interventions are culturally responsive to Black students' needs. Afrocentrism provides Black youth with positive race-based experiences that promote healthy Black identity development (Asante, 2003). When Black children view themselves as centered in Afrocentric values, practices, and structures, their thoughts, behaviors, and emotions are liberated from oppressive Eurocentric ideologies and practices (Lateef et al., 2022). A common practice, like student check-ins during SEL groups, could follow the Afrocentric meeting structure described in *Sisters of Nia* and *Brothers of Ujima* to support Black students. Reintroducing Black youth to Black and African culture through African-centered practices is vital to preventing them from succumbing to harmful Eurocentric perceptions of themselves and to moving toward a strength-based mindset regarding their Black identity (Loyd and Williams, 2017).

### 4.2 Anti-oppressive

Black youth in America are consistently exposed to negative messages, imagery, and oppressive, discriminatory experiences based on racism and anti-blackness (Heidelburg et al., 2022). Additionally, the current sociopolitical climate has increased the race-based hate crimes, speech, groups, and murders of Black individuals (Balaghi and Okoroji, 2023). As a result, SEL for Black children must be anti-oppressive, meaning that it is grounded in critical theories that actively counteract systems of oppression. Critical theories comprise frameworks and ideologies that critique and challenge power structures that oppress marginalized individuals.

Ladson-Billings and Tate (1995) applied critical race theory (CRT) to education as a tool for analyzing race and racism and for critiquing the ubiquitous laws and policies intended to subjugate Black people in the education system. Initially from the legal field, CRT aids in making sense of and responding to systemic racism experienced and endured by Black people (Balaghi and Okoroji, 2023). In education, CRT provides a theoretical framework for understanding Black students' schooling experiences and for investigating how educational institutions perpetuate oppression. McGee et al. (2022) provide several practical strategies for using CRT to support the SEL of REM students and reduce racism and equity barriers in schools. Using CRT allows one to understand how white supremacy is maintained systematically in education through school policies and practices that defend white privilege and protect whiteness at the expense of other racial groups. For instance, colorevasive curricula, the cultural mismatch between teachers and students, deficit-oriented thinking and expectations of Black students, segregation, underfunding, and discriminatory assessments are found in schools to minimize Black students' success (Balaghi and Okoroji, 2023; Ladson-Billings, 1998).

Given the impact of anti-blackness in the American educational system, Dumas and Ross (2016) offer Black Critical Theory, also known as BlackCrit, as an extension of CRT. The authors suggested that BlackCrit is needed since anti-blackness is institutionalized throughout various systems (Dumas and Ross, 2016). BlackCrit helps analyze the prevalence of anti-blackness in education and encourages policy shifts and advocacy to value Blackness. Since BlackCrit aims to promote Blackness, it can serve as a framework for SEL to ensure it is culturally responsive to Black students' needs. Like most children, Black children have multifaceted identities impacted by intersectionality. Since Black children are not monolithic and are multifaceted, SEL must also be rooted in intersectionality. Crenshaw (1989) offered intersectionality as an opportunity to analyze the interconnections among race and other cultural dimensions, such as gender, sexuality, and social class, as well as individuals' experiences of marginalization, oppression, and privilege across their many identities. Black people have a variety of identities that intersect with various systems of oppression that continue to shape and impact the development of Black people across their multiple identities, given the various isms and phobias in society (Dumas and Ross, 2016).

Understanding and applying these critical theories to school-wide policies and practices, such as SEL interventions, is imperative to supporting Black youth (Balaghi and Okoroji, 2023). Failure to address these systems can perpetuate deficit-oriented ideologies and practices that fail to address oppressive structures. To eliminate oppressive school systems, schools could apply one or more critical theories to existing school-wide positive behavioral interventions and supports (SWPBIS) to ensure SWPBIS is responsive to the needs of REM and emerging multilingual students. Johnson et al. (2018) present a framework for implementing culturally responsive SWPBIS to help integrate research-based, culturally competent educational practices into schools. Embedding critical theories to support culturally relevant practices and pedagogy at the core of SWPBIS helps create structures that uplift Black students, reduce discipline disproportionality, and protect against environmental variables that impede students' success. For instance, using an intersectional lens, minoritized students' interests, views, and needs can be incorporated into existing school and classroom policies and practices (e.g., schoolwide expectations and reinforcement systems) to support culturally responsive practices and pedagogy at the core of SWPBIS implementation. Empowering students to participate in establishing norms and expectations in their schools, and incorporating family and community expertise, are ways to center the values of Black populations. McIntosh et al. (2021) aimed to enhance equity in school discipline through culturally responsive SWPBIS. Researchers provided teachers with equity-focused professional development and coaching to help them assess disparities in school discipline and apply culturally responsive practices within a PBIS framework that addresses implicit bias. For example, participants learned to identify equity-focused interventions to meet students' needs and how to implement these strategies within a school-wide approach that aligns with the school's culture. As a result, researchers observed a statistically significant improvement in racial equity in school discipline, further emphasizing the positive impact of culturally responsive PBIS implementation for Black students.

When SWPBIS is responsive to the students it intends to serve, students are provided culturally responsive interventions across each tier that meet their needs. Culturally responsive SEL interventions, rooted in critical theories and embedded across all tiers of SWPBIS, are vital to ensuring that SWPBIS implementation is responsive to the needs of Black students. Furthermore, advocacy is essential to an anti-oppressive implementation of SEL programming. Black students cannot be expected to grow, learn, and thrive in oppressive spaces, meaning SEL programming would be ineffective if implemented in oppressive systems. Hence, BlackCrit can be used to examine schoolwide policies and practices to ensure they do not promote anti-Blackness, impose on Black students' culturally specific expression, values, and behavior. For example, a critical analysis of teachers who are more likely to refer Black students to the office could lead to additional support, coaching, professional development, and accountability mechanisms to ensure equity in how teachers treat Black students. This approach is antithetical to typical data-based decision-making in schools, which focuses on the factors surrounding Black students' perceived behaviors rather than critically evaluating the validity of those perceptions and their impacts on Black students.

### 4.3 Strength-based

Despite the well-known barriers that create inequitable schooling outcomes, what is not often highlighted are the positive outcomes of Black students in education, including the increased number of Black students graduating from high school, attending college, graduating from college, and obtaining advanced, doctoral, and professional degrees (Woods et al., 2023). Although it is necessary to understand the barriers and challenges impeding Black students' success in schools, the overreliance on deficit-oriented educational policies and practices exacerbates Black students' adverse outcomes (Mayes et al., 2022).

For Black students to reach their full potential in schools, SEL must be centered on their strengths. Strength-based empowerment recognizes that Black students, their families, and the community inherently possess strengths and insights that can be built upon to create liberatory educational policies and practices (Albritton et al., 2023; Barnes, 2019). Educators who work with Black students must believe that Black students are capable of educational success and value Black students' unique strengths and brilliance. Centering Black students' strengths in SEL interventions can help educators move away from negative perceptions of Black students. In addition, a strength-based approach can help to achieve SEL that promotes positive educational outcomes for Black children. One way to promote SEL is to center positive racial/ethnic identity development.

Honoring racial/ethnic identity is a prominent aspect of effective SEL to promote social justice and student wellbeing (Mayes et al., 2022). Racial/ethnic identity is how an individual feels connected with and attached to their racial/ethnic group (Belgrave and Brevard, 2015). Researchers have consistently found significant relationships between positive racial/ethnic identity and several developmental outcomes for Black students, including better academic performance (Jones and Lee, 2020), improved self-esteem (Graves and Aston, 2018), and fewer externalizing behaviors (Belgrave et al., 2004). Centering a strength-based approach to SEL enables educators to foster racial/ethnic identity development that celebrates “Black Excellence” by allowing Black students to explore and appreciate the strengths of their identities and racial heritage, thereby feeling empowered (Mayes et al., 2022). Educators can implement SEL interventions that center on the strengths of Black students to support their positive development and equitable outcomes in schools. For example, in addition to behavior-specific praise statements, teachers may choose to monitor the number of affirmations (i.e., positive statements made about someone that indicate respect and caring) they make with Black students. This may directly affect the relationship between teachers and students, influencing various student outcomes. Additionally, two novel culturally enriched SEL interventions, *Black to Success* (B2S; Heidelburg and Collins, 2023) for Black males and *Fix Your Crown, Queen* (FYCQ; Heidelburg et al., 2022) for Black female students, are highlighted as examples.

B2S and FYCQ are novel, culturally enriched, group-based, manualized social skills programs for Black adolescent students. Black school psychologists designed the two programs to aid in the culturally responsive SEL interventions available to Black youth, developed by Black scholars. Both interventions are rooted in one or more critical theories and address aspects of intersectionality. For example, both interventions included Afrocentrism and were designed with an antiracist lens that included a strength-based approach to meet the specific needs of Black youth. In addition, FYCQ utilized the Black feminist-womanist (BFW) framework, which acknowledges the inherited and understudied oppression of Black girls' intersecting identities throughout girlhood and its impact on Black girls' lives (Lindsay-Dennis, 2015). This provides an open space for Black girls to share and articulate their experiences, worldviews, values, and beliefs. Despite experiencing psychological, physical, and social threats in school (Morris, 2007), many Black girls can still thrive in such environments (e.g., developing high self-esteem and achieving academically). Factors identified in BFW research that contribute to the strengths and success of Black girls in harmful and devaluing spaces (e.g., protective factors) were incorporated into the FYCQ curriculum to meet and center Black girls' unique needs.

Both interventions integrate student and family voices, thereby helping to build home-school collaboration. For example, within B2S, conversation cards are used as homework assignments based on the corresponding weekly B2S session. They are intended to provide participants with additional practice in social skills outside the school environment, with families and friends, to further Black males' understanding of the content discussed during each session. Students' experiences completing the conversation cards are also discussed during the individual mentoring sessions embedded within B2S.

Positive racial identity development is at the core of both interventions, which serves as a protective factor for Black youth. Each intervention is culturally enriched to specifically meet the needs of Black students and build positive racial identity. For example, both interventions were strategically designed to be implemented by Black men and women with an Afrocentric focus who are dependable, youth-centered, caring, respectful, and empathetic. B2S explores topics such as masculinity, racism, systemic oppression, navigating being a Black male in the United States, stereotypes and biases, and the media's role in perpetuating harmful stereotypes, all of which are relevant to Black males' positive development (Belgrave and Brevard, 2015). In addition, B2S includes mentoring that provides Black participants with a safe and supervised space to receive support and positive interactions with adult Black men. Mentoring sessions enable Black male youth to share their perspectives and explore aspects of their lives where they may need support. Similarly, the FYCQ curriculum included Black women as interventionists, Black peer models in the SEL groups, and culturally responsive content from the *Sisters of Nia* (Belgrave et al., 2008) to enhance racial identity. Curriculum content included discussions and activities about the Strong Black Woman syndrome, the adultification of Black girls, recognizing negative connotations and stereotypes, and positively restructuring their personal narratives that others may have misunderstood in the past. Scott and Collins (2025) used FYCQ to assess the impact of the curriculum on Black girls' academic engagement, inappropriate behaviors, office discipline referrals, social-emotional competencies, racial identity, and self-esteem. Although there were mixed results, high levels of racial identity were sustained or increased at the end of the program, indicating that FYCQ is a promising culturally responsive SEL intervention for Black girls that emphasizes their experiences and voices within the program.

Positive racial identity protects Black students from the persistent racial trauma experienced from environmental stressors inside and outside of school. The interventions mentioned above are examples of culturally responsive SEL programs that help Black students build their socio-emotional skills and racial identity through liberatory pedagogies and practices that are strength-based and affirm Black students' sense of self-belonging to their racial/ethnic group. Such interventions can be classified as liberatory SEL because they foster Jagers et al.'s (2018) transformative skills of identity, agency, belonging, curiosity, and collaborative problem-solving through culturally relevant content and skills development relevant to Black students.

### 4.4 Student empowerment and healing

For SEL to be liberatory for Black children, educators must first adopt a cultural humility approach by examining the culturally-based implicit biases and assumptions they hold within themselves and the education system (Shriberg and Diaz, 2023). Examining one's cultural attitudes, values, beliefs, and position of power may help to initiate conversations about how school policies and practices are rooted in systemic racism and oppression. Such conversations can cultivate the critical consciousness needed to develop new ideologies and strategies for SEL to meet the needs of the students, families, and communities they are intended to serve.

Critical consciousness focuses on understanding the social and political conditions of the world that aid in the oppression and dehumanization of Black people, as well as the realization that people can change such cruel conditions that REM individuals, particularly Black people, have been forced to experience (Mosley et al., 2021). Sabnis and Proctor (2022) note that critical consciousness increases educators' capacity to perceive the processes and structures that create and sustain oppressive policies and practices within education and encourages a sense of collective action whereby educators work in unity and hold each other accountable to fight collectively against oppression and injustices harming children. Developing a critical consciousness is essential for implementing SEL that empowers Black students; however, it is not enough to think critically and examine biases alone. Educators must take action to bring awareness to inequitable SEL practices and replace them with SEL that facilitates student empowerment and healing.

SEL must promote healing to be liberatory for Black students. Healing involves centering humanization and accepting Black students' full humanity (Ginwright, 2016; Love, 2019). Humanization involves recognizing the importance of being fully human in a world that often subjugates Black people's humanity. Traditional SEL interventions rarely focuses on awareness, consciousness, or critical actions needed to address the oppressive conditions that minimize the wellbeing of Black children (Drake and Oglesby, 2020). Jagers et al.'s (2018) systematic review of SEL interventions in urban schools found that none of the articles addressed racism or its impact on student wellbeing. Camangian and Cariaga (2021) described humanization as the SEL that historically marginalized communities deserve. One example of how SEL can empower youth and aid in healing is Ginwright (2016), who discussed the benefits of a Peacemaker Fellowship Program that focused on outreach and providing services to young men at risk for perpetrating gun violence. The program emphasized that these young men are not beyond repair and highlighted the importance of genuine love, support, and care from positive adult male role models to guide and assist them in making constructive decisions that enhance their lives. For SEL to center healing, educators must fully invest in the full humanity of Black children and allow children the space to belong and the opportunity to heal.

Black children deserve the space to be fully human in schools and question structures and systems imposed upon them (Love, 2019; McCall et al., 2023). Simultaneously, Black children deserve a space that allows them to heal from continuously experiencing systems that dehumanize them. SEL interventions must invite, welcome, and center all aspects of Black students' lived experiences (Lin et al., 2023). Centering healing in SEL interventions extends beyond solely teaching coping skills such as relaxation strategies. Instead, healing aims to allow students to be human and develop knowledge of self through culturally responsive practices and ideologies that help Black students learn about who they are, where they come from, and their value to the world (Ginwright, 2016). For instance, restorative practice community-building circles can be used with SEL to create a supportive, positive, and inclusive environment for Black youth. Community-building circles are a powerful way to provide individuals with a space to unite, share their thoughts and feelings, and build positive relationships. Engaging in community-building circles can foster a sense of community and help Black youth strengthen their identity, agency, belonging, curiosity, and collaborative problem-solving, while reducing exposure to exclusionary discipline (Darling-Hammond, 2023).

Safe spaces help to prevent the constant spirit-murdering of Black students and families (Simmons, 2021) and provide an opportunity for healing where Black students' dignity can be restored, their wellbeing can be nurtured, and their Black lives can truly matter (Ginwright, 2016; Love, 2019; Mayes et al., 2022). Therefore, educators must commit to implementing and sustaining culturally affirming spaces that ensure Black students' mental and physical safety and wellbeing in schools. Belgrave et al. (2000) conducted an SEL intervention with Black girls that included an overnight retreat and weekly sessions over 4 months to build strong relationships among participants and interventionists, as well as to promote self-confidence, self-esteem, and gender and cultural identity among the girls. During the intervention, participants engaged in conversations and healing-centered practices such as creative dance, movement, songs, sign language, and other art forms to address topics relevant to Black girls, including hygiene, hair, health, nutrition, and positive body image. When space is provided for healing in SEL interventions, Black students can foster hope and school belonging to support their wellbeing. In a world that often diminishes Black students' hope and wellbeing, Black students may disinvest in their academic success as a response to an unsupportive educational culture (Balfanz et al., 2014). Therefore, SEL should center on healing to restore Black students' hope and wellbeing. When Black students have space to heal, they can choose resistance rather than resilience, which allows them to engage in intentional critiques and confront oppression rather than normalizing inequities and discrimination and placing the onus on students to be resilient and cope with oppression (Jordan et al., 2024). When Black students have hope, they can dream about their future goals and take the necessary steps to achieve them. Promoting hope through SEL that centers healing also helps Black students to feel connected to and valued by their school community. When Black students have a sense of hope and belonging in school, research increased motivation, academic engagement, perceived value of school, and overall wellbeing (Dixson and Gentzis, 2023).

In addition to the space to heal, Black youth should experience SEL interventions that allow them to demonstrate their agency and voice. For SEL to liberate Black youth, SEL must promote student empowerment by providing safe spaces that allow Black children to be human. Compared to their peers, Black students have a lower perception of cultural acceptance and adult social support while attending school (Adams and Roach, 2024; Collins et al., 2023), despite Black youth often endorsing seeking social support or talking to someone to cope with racial discrimination as one of the most used coping strategies (Griffin et al., 2020). A liberatory approach to SEL centers students in all aspects of intervention delivery, including the selection and measurement of target behaviors, goal setting, co-creating expectations, co-constructing intervention content, and more. Empowering Black youth to make educational decisions and contribute their energy and leadership to developing and implementing an intervention counters the adult-centered, authoritarian systems they often navigate. Estrapala and Greishaber (2022) identified numerous empowering strategies that can be implemented to improve students' self-determination in behavioral and social-emotional interventions and reduce contact with exclusionary discipline, including giving them space to think about how their current behaviors are connected to their future goals, reviewing data with students, and improving their self-awareness about their behavioral triggers and potential replacement behaviors. Black students have reported that improving their self-determination skills is related to their motivation in school, time management, and teacher support, indicating that student agency is essential to culturally relevant intervention (Parker et al., 2020). Self-determination (i.e., *Kujichagulia*) is one of the seven principles of Karenga's (1988) *Nguzo Saba*, underscoring its importance to Black culture and to the Black community. As such, Black youth should inform and co-construct SEL interventions, ensuring that their voices are heard in the procedures and outcomes of the intervention.

### 4.5 Evidence-based practices

Evidence-based practices “integrate the best available research with clinical expertise in the context of patient characteristics, culture, and preferences” (American Psychological Association Presidential Task Force on Evidence-Based Practice, 2006, p. 273). Educators may enact practices that are not research-based or rooted in evidence-based principles. Engaging in such practices does a disservice to students and the role of health service professionals and educators. Although educators' roles are multifaceted, and the need to stay up to date with evolving best practices can seem impossible at times, educators must identify, adapt, implement, and evaluate high-quality, evidence-based practices. Educators play a fundamental role in integrating cultural competence, knowledge, and appreciation into their work through targeted interventions that support children in school. Kratochwill and Shernoff (2004) suggest that an intervention should be considered evidence-based when information about its contextual application is specified and it has shown efficacy in practical implementation and evaluation. Shapiro (2011) highlights guiding principles for interventions, suggesting that evidence-based practices are grounded in theory and research and include efforts to promote generalization and maintenance; decision-making through progress monitoring; opportunities to respond; repetition; positive reinforcement; immediate corrective feedback; and practical models/prompts. Kratochwill and Stoiber (2002) provided a checklist of several considerations for EBIs, including:

*Does your client appear similar to those described in the EBI? Are the implementation conditions for the EBI similar to those in your setting? Was an ongoing evaluation (repeated assessment) of student progress conducted? Can individual student characteristics be identified that are related to intervention outcomes? Have the EBI positive effects reported in research been replicated with your student(s)? Have you or others in your school setting replicated the EBI more than once? Do you plan to adopt the EBI for future implementation in your setting?* To avoid potential harm to students, educators can use Kratochwill and Stoiber's (2002) checklist or other related resources (e.g., Norcross et al., 2017) to immediately abandon interventions that demonstrate little to no effectiveness, have limited support in applied settings, or are inapplicable to their students.

Fallon et al. (2022) reviewed the characteristics of school-based SEL interventions and found that the most substantial intervention effects were in studies that included evidence-based practices and adapted interventions. Evidence-based practices are essential for supporting Black students' positive youth development and liberation. Evidence-based practices that consider the socio-ecological contexts that impact Black students can help liberate Black children. For instance, Marraccini et al. (2022) suggested a trauma and justice, equity, diversity, and inclusion (JEDI)-informed approach to suicide prevention for Black males in schools. This approach considers the socio-ecological contexts impacting Black males and is culturally grounded, with prevention and interventions across a multi-tiered system of support that prioritize Black males' lives.

Additionally, for Black children who are exposed to violence or at risk of experiencing violence, Case (2017) and Cohen et al. (2006) highlight evidence-based psychosocial interventions that help youth navigate social-structural adversity in their lives. For instance, trauma-focused cognitive behavioral therapy (TF-CBT) is presented as a treatment to target trauma-related symptoms such as PTSD, depression, anxiety, and trauma-related shame, and cognitions such as self-blame (Cohen et al., 2006; de Arellano et al., 2014). Race considerations for TF-CBT have been empirically studied across diverse youth populations, supporting its cultural utility (Cohen et al., 2006). Integrating or coupling TF-CBT in SEL can be liberatory for Black youth exposed to violence as well as for the caregivers, as TF-CBT also includes a caregiver component that aims to enhance parental support, decrease emotional distress, and improve positive parenting practices (de Arellano et al., 2014).

It is important to note that, despite practices being deemed evidence-based and effective, they may not be effective for REM students due to a lack of cultural considerations. Graves et al. (2021) found that none of the evidence-based behavioral interventions in the What Works Clearinghouse disaggregated results by race or included cultural adaptations, further supporting the idea that the criteria for evidence-based interventions in the field often do not account for race and other identities. Jones et al. (2026) highlight issues of race and racism, as well as challenges to the generalizability of SEL interventions by evaluating the existing evidence base for CASEL SELect interventions across diverse racial groups. Researchers suggest that evidence of SEL efficacy for specific REM groups is lacking in the current literature, underscoring the need to analyze race in SEL research to generate evidence of effectiveness for REM groups. Therefore, educators must include Black students in the evidence gathered on SEL interventions and consider the cultural and personal match between evidence-based practices and the characteristics, values, and beliefs of the students they intend to serve (Floyd and Norfolk, 2023).

The practice-to-research gap in social justice interventions in school psychology indicates that research is lagging behind advocacy work in the field. As such, SEL must be implemented with the best available evidence of effectiveness for Black populations and grounded in theories of Black youth development and identity. Since evidence-based practices do not follow a one-size-fits-all approach, educators can adapt interventions to fit their students' lived experiences and the needs of the setting. (Arora et al. 2025) introduced the cultural adaptations content checklist (CACC) to encourage the use of cultural adaptations in evidence-based psychological interventions for REM youth. The CACC can be a valuable tool for enhancing the cultural and contextual relevance of evidence-based SEL interventions to better outcomes among REM youth.

## 5 Systemic school changes for practice

Incorporating these components into schools' practices will require significant changes to systemic educational policies and practices, which may seem impossible. However, this work is worth doing, and these changes are possible with conviction, leadership, self-reflection, student-centered values, and critical action. To create truly safe, affirming, and liberatory educational spaces for Black youth, educators must honor their commitment to ensuring safe and supportive school environments and work to reform the current education system to reimagine education in ways that reflect the values of the Black community. To foster systemic change and promote social justice, Table 1 offers recommendations for implementing the five components at the system level to enhance individualized interventions, build community partnerships, and develop systemic policies and practices that serve the needs of Black youth. The recommendations can help bridge the gap between school-home/community collaboration and support systemic changes to school-based SEL implementation that empower Black students.

**Table 1:** Systemic changes to promote SEL for Black students.

Component	Individualized school interventions	Community collaboration	Systemic policies and practices
**Afrocentrism**	•Incorporate Afrocentrism into schoolwide and classroom interventions to promote the use of culturally responsive supports.	•Engage community leaders as Afrocentric mentors to promote school-community collaboration.	•Create policies to promote Afrocentric pedagogy and practices within schools.
See Belgrave et al. (2008, 2011) and Lateef and Anthony (2020) for examples			
**Anti-oppressive**	•Review discipline policies and practices using critical theoretical frameworks to eradicate unjust policies and practices. •Ensure educators engage in ongoing professional development and self-reflection for biases and oppressive practices.	•Engage community members in conversation regarding existing school policies and practices.	•Dismantle oppressive policies that maintain oppression and white privilege. •Review federal and state policies using critical theoretical frameworks to minimize whiteness. •Recognize racial harm to eradicate unjust policies and practices. •Fund schools equitably to assist in providing SEL interventions.
See Johnson et al. (2018), McGee et al. (2022), and Turner et al. (2022) for examples			
**Strength-based**	•Use SEL practices that promote safety and inclusivity. •Offer students choices on how to be acknowledged. •Strength-based assessment to inform SEL implementation.	•Engage with community members regarding strengths and areas of improvement for Black students.	•Advocate for strength-based policies and practices to promote equitable school outcomes.
See Case (2017); Clonan-Roy et al. (2016) and Heidelburg and Collins (2023) for examples			
**Facilitating student empowerment and healing**	•Implement SEL interventions that promote healing. •Empower youth voice in SEL interventions and universal practices.	•Create authentic partnerships to promote school-community collaboration. •Develop opportunities through SEL for supportive, trusting relationships with the community.	•Ensure Black students' identities are affirmed and represented throughout pedagogical practices and policies. •Empower Black students to co-design socially just school culture policies. •Eliminate anti-black oppressive policies.
See Belgrave et al. (2000); Darling-Hammond (2023) and Ginwright (2016) for examples			
**Evidence-based**	•Promote the use of culturally responsive, strength-based, and evidence-based interventions. •Engage in data collection to assess the use and effectiveness of SEL. •Assess the attitudes toward the adoption of culturally responsive SEL.	•Collaborate with youth, caregivers, and community members regarding SEL practices for Black students.	•Advocate to school leaders regarding unjust policies and practices. •Promote policies and practices to ensure evidence-based SEL interventions are used and support equitable outcomes.
See Graves and Aston (2018), Jones and Lee (2020), and Scott and Collins (2025) for examples			

## 6 Conclusion

In its current form, color-evasive SEL interventions risk advancing white, middle-class ideals, standards, and ways of being, knowing, and feeling. As such, this can result in compounded harm and discipline disproportionality for Black youth who navigate oppressive educational systems. In contrast, this scholarship catalyzes forward-thinking research and innovations in SEL to ensure equitable outcomes for Black students. We have proposed SEL components that are foundational to supporting culturally responsive SEL for Black students. These components transform SEL into a vehicle for hope, liberation, and, ultimately, the advancement of the Black community. We have also highlighted research on promising practices that advance liberatory SEL for Black students.

A strength of this scholarship is the positionalities of the authors. The first author is a Black/African American man, a Nationally Certified School Psychologist, and an Assistant Professor of School Psychology at an urban, research-intensive, community-engaged institution. He identifies as a Black scholar-activist who researches cultural adaptations of evidence-based, individualized, and systems-level interventions for Black students, particularly Black males, in education. His research and lived experience as a Black man, scholar, and product of lower-SES environments and the US public education system have shaped his passion and research on creating change that leads to the liberation of all Black people in all spaces. The second author is a Black, American, heterosexual, cisgender woman who is an Assistant Professor of School Psychology at a research-intensive university in the South. While receiving her primary education in the Northeast, she was among the few Black students in a predominantly white school district (less than 5%). She witnessed the implementation of colorblind approaches for academics and behavior management in schools. Therefore, her research is informed by a commitment to dismantling the systems of oppression embedded in the school setting and to embracing Black youth's cultural identity through culturally responsive practices and community engagement. The third author is a Black, cisgender, heterosexual man who serves as a university administrator and Professor at a large urban institution. Growing up in the South exposed him early to the detrimental effects of racism and economic marginalization. His work is rooted in these experiences, as he brings this perspective to his writing, leadership, graduate student preparation, teaching, community work, and now university administration. All authors hold at least one marginalized identity, possess multiple advanced degrees and certifications, and are passionate about improving equitable outcomes for minoritized students, particularly Black students.

## 7 Implications

The proposal of five key components for implementing school-based SEL to empower Black youth has two significant implications: (1) catalyzing research that can liberate SEL for Black populations and (2) advancing practical applications of school-based SEL programs to redress oppressive educational systems and anti-black practices. In catalyzing research, we aimed to highlight the current barriers impeding the positive social-emotional development of Black youth, thereby providing further evidence of the need for key components of school-based SEL to support Black youth. We provided critical foundational components for school-based SEL implementation that center on the needs of Black students. Although the highlighted components are not the only elements that can support SEL implementation for Black students, they serve as a foundation for further investigation to support students who warrant such support. We also discussed liberatory SEL interventions, such as *Fix Your Crown, Queen*, and *Black to Success*, that are specifically designed to support Black students. Researchers are encouraged to replicate these studies, use data to inform improvements to these and other programs, and build new interventions from the ground up with Black students at the center.

For advancing practical applications, we encourage practicing teachers, administrators, school psychologists, counselors, and other educational support staff to use the highlighted research to inform school policies and practices that lead to better outcomes for Black students. Given that U. S. educators are mostly white, even in schools and districts where REM youth constitute the majority of the population (National Center for Education Statistics, 2023), educators must first engage in deep, critical reflection and build their multicultural competence and critical consciousness to effectively implement these five components and transform SEL into a vehicle for hope and liberation for Black youth. Diemer et al. (2016) outline traditional and contemporary approaches to fostering critical consciousness and ways to assess it. In addition, McDowell et al. (2026) highlight that culturally responsive implementation begins with educators reflecting on their cultural identities, privileges, biases, and life experiences. Researchers emphasize the importance of systems and structures that support sustained commitment to continuous professional development and critical self-reflection to address personal biases that contribute to inequities in educational settings. Engaging in such practices provides educators with opportunities to build their knowledge, awareness, appreciation, and skills to better support REM youth. Once educators develop their critical consciousness and multicultural competence, they are better equipped to implement culturally responsive interventions and supports or to partner with communities of color, regardless of their cultural identities.

Given the ongoing legislative and policy issues regarding what can and cannot be implemented in schools to support diverse students, educators must strengthen the partnership between the Black community and schools to ensure that Black students receive culturally responsive SEL interventions. Community-based organizations and educational spaces have played a significant role in supporting children, especially those marginalized by racism and anti-Blackness (Baldridge et al., 2024). Community spaces provide coordinated academic and youth development opportunities outside school hours, such as before and after school or on weekends, and intentionally offer youth spaces for healing-centered engagement, culturally responsive mentorship, and equity- and justice-centered practices that foster belonging and identity Baldridge et al., (2024). (McGovern et al. 2026) describe practices used in out-of-school programs to support Black youth's racial-ethnic identity and sociopolitical development. Researchers found that such programs incorporated culturally and developmentally relevant content that countered deficit messages; provided activities that helped youth develop positive identities and explore how historical harms shaped their lives; fostered safe, positive environments and relationships; connected youth to the cultural Diaspora and the historical legacy of individuals who resisted marginalization and oppression; and supported youth in building and practicing skills that could contribute to their success and prepare them to take action toward a more socially just future.

Schools should partner with communities to ensure that SEL interventions meet youth needs as the community deems appropriate and to advance SEL interventions free of white supremacist and deficit narratives about Black youth. For example, joining coalitions, attending local council meetings, and consulting with administrators and district lawyers on legislation can strengthen partnerships between the community and schools, helping to provide support aligned with youth needs. In addition, the proposed components and interventions should be adapted to the local context; practitioners are encouraged to tailor these components to their schools and to advocate for SEL interventions that support Black students. Using SEL to address both intentional and unintentional harm will help build communities of support for Black students.

## 8 Future directions

The most immediate future directions are research studies examining the effectiveness of Liberatory SEL interventions for Black youth. Researchers should investigate culturally responsive SEL interventions, such as *Fix Your Crown, Queen*, and *Black to Succes*s, as well as others like *Sisters of Nia* and *Brothers of Ujima*, to center Black students' liberation. Other studies may focus on culturally adapting SEL interventions with Afrocentric components (e.g., drumming, libation, Afrocentric values, community and family involvement) to enhance their effectiveness. Studies may focus on cultural adaptations of SEL interventions for specific populations within the Black community (e.g., Black students with dis/abilities, Black LGBTQIA+ students, and Black immigrants to the United States) to ensure the cultural relevance of these interventions within each community.

Another future direction involves a culturally responsive assessment approach of various skills and behaviors among Black students. Assessment is a crucial element to determine the effectiveness of interventions and supports. Implementing a SEL intervention without related culturally responsive assessments may result in ineffective, deficit-minded, and potentially harmful decision-making about student outcomes. As such, researchers should identify the components of liberatory assessment in Black youth. This may involve strengths-based assessment, cultural competence of assessors, cultural relevance of assessment tools, student agency in selecting goals and assessments, and other components. In the psychological literature, more attention should be paid to assessing essential variables for Black youth, such as racial/ethnic identity, acculturation, self-esteem, and critical consciousness. As interventions are developed to address these concerns, guidance should be provided on how much these variables are expected to change in response to the dosage and duration of the interventions. Assessment should also focus on qualitative methods for data collection in research and practice, and provide guidance on best practices in decision-making, technical adequacy, and the trustworthiness of qualitative data within Black populations.

Given the push to ensure EBIs are responsive to the needs of REM youth, it is important that Black voices are centered and that EBIs are not anchored in theories and practices that fail to recognize Black youth as human, agentic, and situated within systems. Community participatory action research that centers youth participatory action research (YPAR) can be a crucial approach to support research on SEL interventions that are culturally responsive to the needs of Black youth. It can also be used to collaborate with youth and communities to collect data to understand and build evidence on which SEL interventions are best suited to meet Black students' needs.

To truly advocate for Black youth and their social-emotional needs, researchers should use YPAR projects to lead to outcomes that support Black youth, such as increased advocacy skills for students and adults, development of positive ethnic identity and sense of purpose, enhanced individual and collective efficacy, and increased school bonding, social networks, and supports (Ozer et al., 2010). Nash et al. (2024) assessed the evidence for YPAR as a means of promoting youth SEL outcomes and found a growing body of YPAR literature supporting youth development outcomes, suggesting that YPAR is a promising approach for promoting positive youth transformative social-emotional learning. YPAR projects have improved social, emotional, and academic outcomes for REM students and typically result in policy recommendations that can lead to transformative systemic changes in educational practices (Bertrand and Lozenski, 2023). Cohen et al. (2020) highlight how YPAR can inform school districts' educational policies and decision-making, further providing evidence that YPAR can support decision-makers' perspectives. Researchers encourage policymakers to develop additional policies that facilitate YPAR in education and to create policy structures that support YPAR in informing school policies and practices. Moreover, researchers can continue to explore the social-emotional needs of Black youth by engaging them as co-researchers in projects that examine and recommend changes they would like to see in SEL implementation in their schools.

A final future direction involves the professional competencies that school psychologists and other professionals need to establish liberatory education models for Black youth. For example, educators can use Turner et al. (2022) black love, activism, and community (BLAC) model of healing and resilience to promote Black activism that centers love and community, thereby supporting liberatory practices for Black youth. Research should inform the professional development needed for individuals to advocate and make changes in oppressive systems. Studies can also guide self-reflection on one's own experiences, biases, and intersectional identities, as well as on how to encourage this work among our colleagues and community partners. Systems-level advocacy is also critical to advancing the policies and practices that shape Black students' experiences in schools, as implementing culturally responsive SEL interventions without changing systems and policies is akin to building a house of cards.

As we examine our nation's deeply rooted issues of racism and oppression that limit the liberation of Black children, we must recognize that current school policies and practices often serve to uphold whiteness, protect white privilege, and sustain white supremacy. We hope that educators and health service professionals will use this information to become agents of change, advocating for and ensuring equitable outcomes for Black children through SEL.

## Data Availability

The original contributions presented in the study are included in the article/supplementary material, further inquiries can be directed to the corresponding author.
